# Qualitative investigation of skills and knowledge required for job readiness in newly graduated speech-language pathologists

**DOI:** 10.1371/journal.pone.0354088

**Published:** 2026-07-21

**Authors:** Madhawi K. Altaib, Aseel Alkadhi, Ghada T.A. Najmaldeen, Lamya Aldukair

**Affiliations:** Rehabilitation Health Sciences Department, College of Applied Medical Sciences, King Saud University, Riyadh, Kingdom of Saudi Arabia; Manipal Academy of Higher Education, INDIA

## Abstract

This study explores the critical skills and knowledge that new speech and language pathologists (SLPs) must acquire to transition successfully into the workforce. While university programs aim to prepare students adequately, gaps remain in identifying the specific competencies essential for job readiness. This study employed a qualitative research approach, conducting focus group interviews with key stakeholders, including alumni, faculty, clinicians, and employers. Data were analyzed using thematic analysis. A total of 15 participants took part in five focus groups. The analysis identified four main themes: *essential personal characteristics, exhibiting independence in managing work, foundational academic knowledge, and general clinical skills for SLPs*. The findings emphasize the importance of traits such as a positive attitude, flexibility, and the ability to analyze situations and make informed decisions. Additionally, the study highlights the need to expand clinical training and integrate a more specialized curriculum. While some advanced topics require further exploration, they may be too complex for entry-level professionals without additional experience. Providing students with exposure to diverse clinical settings and work environments during training is also crucial. To better prepare newly graduated SLPs, university curricula must evolve to equip graduates with the necessary skills and knowledge for varied clinical settings and to meet the profession’s growing demands.

## Introduction

The allied health sciences education and training field has faced considerable challenges in ensuring that graduates acquire the fundamental knowledge and clinical skills required for effective workforce readiness. [1,2]. Speech and Language Pathology (SLP), in particular, demands the integration of complex knowledge and skills to assess, diagnose, and treat disorders related to speech, language, social communication, cognitive-communication, voice, and swallowing across the lifespan of both children and adults [3,4]. To meet this goal, curriculum design needs to address evolving healthcare demands, shifting demographics, and expanding professional roles while fostering graduate competencies in clinical, interprofessional, and ethical practice [5]. As such, understanding how well academic programs prepares graduates for practice and where gaps exist has become a key focus of research.

On an international scale, several studies investigated the readiness of newly graduated SLPs for the workforce. For example, in New Zealand, Tillard G, Lawson K, Emmerson S. [6] examined the views of SLP graduates and their employers on how well one university program prepared graduates for the workforce. They used a written survey that included rating graduates’ skill levels and open-ended questions addressing areas for improvement and professional support and development. Their findings identified topic areas of weakness and clinical management skills requiring more focus, such as caseload prioritisation, teamwork and time management. In addition, they showed that employers and graduates sometimes differed in their rating of specific skills. More recently, a qualitative study in Australia investigated supervisors’ perceptions of SLP graduates regarding the essential skills for work readiness. General professional skills extending beyond technical skills were identified by supervisors as crucial for graduates to succeed in work. Those skills include teamwork, continuing learning, independence and attitude [7]. Similarly, studies from the United States investigated readiness in specific clinical domains, reporting limitations in graduates’ preparedness and confidence across areas including AAC [8], dysphagia [9], literacy [10], and traumatic brain injury [11]. Collectively, this body of evidence highlights a gap between university training and the diverse, evolving demands of clinical practice, underscoring the need to clearly define the competencies required for effective professional performance

Despite this growing international evidence, little is known about the competencies required for SLP practice in Saudi Arabia. The first SLP program in Saudi Arabia was established in 1979 at King Saud University. Over the past ten years, multiple universities have also started offering regional SLP programs. The increase in the number of programs offering Speech and Language Pathology degrees aligns with the rise in market demand in the Kingdom of Saudi Arabia and increasing awareness of the population’s need for services.

The curriculum of most speech-language pathology programmes includes similar course distributions, which are benchmarked against international curricula from the USA, UK, Canada, and Australia. Additionally, all graduates must complete an internship to be licensed as SLPs [12]. Although these programs are benchmarked against international curricula, the previously identified gaps in graduate readiness raise concerns about whether such models adequately address local workforce needs. To ensure that graduates develop the necessary skills, knowledge, and attitudes to become competent professionals, speech-language pathology university programmes must align with stakeholders’ requirements. This alignment is essential for meeting the demands of the professional environment and the expected standards of speech-language pathology graduates [13]. This requires us to consider the unique professional environment of speech-language pathologists in Saudi Arabia, including regulatory policies, the demand for speech-language pathology services, and the rapid societal changes. For example, there are shortages of speech-language pathologists and an inequitable distribution of facilities, as services tend to be available in major cities [14]. According to Khoja and Sheeshah, the ratio of SLPs to population in Saudi Arabia was 0.67 per 100,000 [14]. A study conducted by Alanazi among SLPs, revealed that 81% of respondents stated that the number of SLPs in clinical settings is insufficient to meet the existing demand for SLP services [12]. These workforce challenges further highlight the need to clearly define the competencies required for effective SLP practice within the Saudi context.

Importantly, this need aligns with the goals of Saudi Vision 2030, particularly the Human Capability Development Program, which emphasizes aligning educational outcomes with labor market needs, adopting competency-based approaches, and developing a highly skilled healthcare workforce [15–17]. Within this framework, higher education institutions are expected to ensure that graduates possess the knowledge, clinical skills, and professional attributes needed to meet national priorities and workforce demands. Therefore, this study aims to identify the necessary knowledge, clinical skills, and attitudes required to develop competent speech-language pathologists who serve clients with various communication disorders in Saudi Arabia. It is considered one of the first to investigate the unseen gap between what university programs’ curricula offer and market demands in Saudi Arabia. Thus, it contributes to ongoing national efforts to enhance workforce readiness, support healthcare system development, and advance outcome-based education in line with Vision 2030 objectives. In addition, it responds to international evidence highlighting mismatches between academic preparation and workforce expectations, contributing to a more nuanced understanding of how competency frameworks can be adapted to suit diverse healthcare and educational environments.

## Methods

The study aimed to investigate the necessary skills and knowledge that speech-language pathology graduates should possess. This research used a qualitative study approach involving focus group interviews with three main stakeholders. Qualitative research methods are ideal for exploring viewpoints and understanding stakeholders’ perspectives [18]. Researchers used a constructivist qualitative approach in similar studies, emphasising the value of participants’ views and experiences in understanding the skills and knowledge required by speech and language pathologist graduates [19,20]. Furthermore, this study utilised focus group interviews because they permit researchers to understand people’s beliefs and experiences in a natural setting where social context plays a more vital role than personal accounts [21 old 20].

The research team comprises four female faculty members from the Speech and Hearing Therapy program who are qualified PhD holders, SLPs and advocates for the Speech and Language Pathology field. The King Saud University Human Research Ethics Committee evaluated and approved this qualitative study (Approval No. KSU-HE-22–270), with an approved extension (Approval No. KSU-HE-23–738).

### Study setting and sample

Our study used purposive sampling to conduct focus groups with three distinct stakeholder groups: the Speech and Language Pathology program alumni, program faculty members, and employers who have hired program graduates.

An invitation email was sent to a sample of SLPs working in different settings in Saudi Arabia, targeting a wide range of experience and those familiar with the current program(s). The email included detailed information about the study purpose and focus group protocol. Informed consent was obtained electronically through an online survey prior to participation. Only participants who provided consent were able to proceed to the demographic questionnaire, which collected information on age, gender, employment status, and educational background. Data collection was conducted between October 2022 and September 2023.

The initial interview guide was drafted by two members of the research team based on the study objectives and previous literature addressing similar topics in allied medical sciences education and professional preparation [19,20]. The questions were designed following Krueger and Casey’s five categories, which are proven to facilitate the flow of a focus group. These categories include Opening, Introductory, Transition, Key, and Ending [22]. The draft questions were subsequently reviewed by all members of the research team to assess clarity, relevance, and alignment with the study aims. Minor revisions were made through collaborative discussion and consensus among the researchers, including refinement of question wording and sequencing to improve the flow of the focus groups. The interview guide was also pilot tested with individuals familiar with the field of speech-language pathology to evaluate question clarity and appropriateness prior to data collection. The interview guide can be found in Appendix 1 in supplementary material.

All focus group interviews were conducted online (using Zoom videoconference software) because all participants had work obligations that made attending in-person meetings difficult. Participants were asked to attend the interviews from a private location and were reminded not to share discussion content outside the group to support confidentiality in the online setting. The 2^nd^ author (AA) was the focus groups moderator who is a faculty member with working experience in curriculum and internship committees and received training in conducting qualitative research. To maintain consistency across interviews across focus groups, the same research team member moderated all focus groups, while another research team member attended all sessions to take field notes. All interviews were conducted in Arabic, transcribed verbatim in Arabic, and subsequently translated into English for reporting purposes. All members of the research team were bilingual in Arabic and English, and translations were reviewed collaboratively to preserve meaning, contextual nuances, and analytic accuracy. The interviews lasted approximately one to two hours. Participants were free to discuss any pertinent topics not covered by the guide.

To ensure that each participant could express their experiences and viewpoints, separate focus group interviews were conducted with each group. Specifically, two focus groups were conducted with alumni, one with faculty members and two with employers. This allowed us to gather a wide range of perspectives from each group, which helps understand this program comprehensively. Since the research team consisted of faculty members within the Speech and Hearing Therapy program, some participants may have been familiar with members of the research team through prior academic or professional interactions. To reduce potential social desirability bias, participants were informed that their responses would be treated confidentially and used solely for research purposes. They were also encouraged to express their views openly and reassured that there were no right or wrong answers. Additionally, interview questions were phrased in a neutral and non-judgmental manner to minimize pressure to provide socially desirable responses. Interviews continued until data saturation was achieved. Saturation was assessed collaboratively by the research team through regular discussions of emerging codes and themes during ongoing data collection and preliminary analysis. No new themes emerged in the fourth focus group; however, one additional focus group was conducted to confirm that saturation had been reached.

### Data analysis

**Fig 1:**
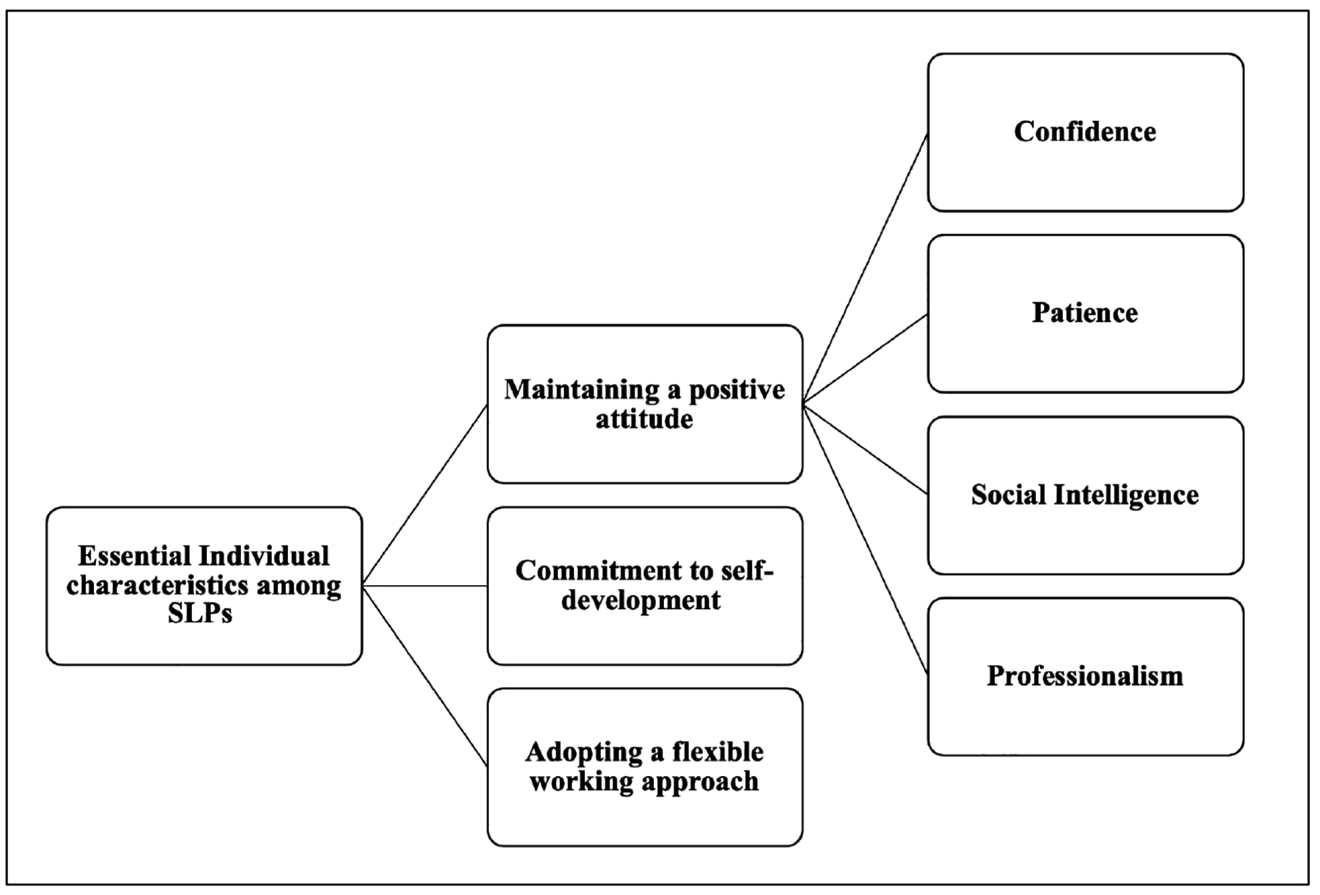
Original illustration developed by the authors demonstrating the qualitative coding and theme development process used in the study.

All five interviews were audio recorded using Zoom software and analysed using reflective thematic analysis [23]. A research assistant transcribed the recordings, and MA and AA reviewed all transcriptions to ensure accuracy. Atlas software was used to support the analytic process, which involved several steps: familiarising with the data, generating initial codes, sorting and grouping codes to identify potential themes, and reviewing, defining, and interpreting final themes [23]. During the initial phase, both authors (MA and AA) read all interview transcripts and formed their initial interpretations. MA then independently coded two transcripts, while AA coded the remaining three. The two researchers subsequently reviewed each other’s transcripts and codes independently, before meeting to discuss any discrepancies. Any differences in coding or interpretation were resolved through reflective discussion and re-examination of the original transcripts until consensus was reached, ensuring consistency in the development of themes and subthemes. The codebook was developed iteratively throughout the analytic process, informed by repeated engagement with the transcripts and ongoing discussion among the research team. The primary themes were then created by grouping codes to establish subthemes. Themes and subthemes were not retained based on a predetermined minimum number of quotations or frequency counts. Consistent with a reflective thematic analysis approach, themes were developed based on the relevance, meaning, and patterned significance of the data in relation to the research questions. The entire team discussed and identified all topics and sub-themes to improve the accuracy and dependability of the study results [18]. Refer to Fig 1 for an original illustration developed by the authors demonstrating the qualitative coding and theme development process used in this study, refer to Appendix 2 for the codebook.

## Result

Five focus group interviews were conducted with 15 participants (13 female and two male). One participant from the alumni group did not attend the focus group session due to an urgent meeting. The participants were divided into three groups: alumni (n = 7), faculty members (n = 4), and clinicians or employers (n = 4). Participants represented a range of clinical and academic settings across Saudi Arabia, including hospitals, private clinics, long-term care facilities, and university settings. Employers and faculty members also reflected varied clinical subspecialties, including pediatric, adult communication disorders and fluency disorders. This diversity allowed the study to capture perspectives from different service delivery environments and professional roles, refer to Table 1 for the characteristics of the participants.

**Table 1:** Characteristics of the study participants (N = 15).

Study Participants		ALUMNI N = 7	FACULTY N = 4	EMPLOYERS N = 4	TOTAL N = 15
**EDUCATION LEVEL**	**Bachelor**	7	0	0	7
	**Master**	0	3	4	7
	**PhD**	0	1	0	1
**AGE**	**21-29**	7	0	0	7
	**30-39**	0	2	4	6
	**40-49**	0	2	0	2
**GENDER**	**Female**	7	4	2	13
	**Male**	0	0	2	2
**WORK EXPERIENCE**	**Less Than One Year**	7	0	0	7
	**11-15**	0	2	4	6
	**16-20**	0	2	0	2
**WORK SECTOR**	**Hospital**	3	0	3	6
	**Private Clinic**	3	0	1	4
	**Long Term Care Facilities**	1	0	0	1
	**University**	0	4	0	4
**SUBSPECIALITY**	**Pediatric Communication Disorder**	0	1	2	3
	**Adult Communication Disorder**	0	1	2	3
	**Fluency disorder**	0	1	0	1
	**Not subspecialized**	7	1	0	8

The analysis of the five interviews revealed four main themes: *Essential individual characteristics among SLPs, exhibiting independence in managing work, academic knowledge for SLPs, and general clinical skills for SLPs.* Each theme had several subthemes, as illustrated in Table 2.

**Table 2:** Focus group interviews identified themes and subthemes.

Theme	Subthemes
Essential Individual characteristics among SLPs	Maintaining a positive attitude, adopting a flexible working approach, and commitment to self-development
Exhibiting independence in managing work	Demonstrating analytical skill for SLP, decision-making ability, and exhibiting a strong sense of responsibility
Academic knowledge for SLPs	Foundation knowledge essential in SLP curriculum, speciality knowledge for SLP curricula and topics on current issues in SLP curricula
General clinical skills for SLPs	Core skills to deliver services for clients and general work competencies for clinicians

### Theme 1: Essential individual characteristics among SLPs.

Participants stressed that graduates must possess specific characteristics essential for effective SLP practice. Clinicians work with clients with various communication disorders, their caregivers, and other professionals. These characteristics were grouped into three subthemes: maintaining a positive attitude, adopting a flexible working approach, and commitment to self-development.

**The first subtheme, maintaining a positive attitude,** refers to the characteristics and attributes that enable graduates to maintain a positive attitude while working with clients and other professionals in multidisciplinary teams. This includes professionalism, which participants from all groups highlighted. It was agreed that SLPs are expected to show respect in their interactions and maintain professional conduct in all aspects of behaviour. As one of the participants in the employers’ group stated, this also includes dressing appropriately.“The Code of Ethics includes many things, whether at the level of personal conduct or communication or even appearance, I mean how the specialist interacts with patients or caregivers. Although it’s not easy to measure, it’s essential to maintain high levels of professionalism to ensure customer satisfaction” (Employer group −2). The “maintaining a positive attitude” subtheme also includes personal qualities such as empathy and patience, which are essential as SLP is considered a humanistic profession that puts the client at the centre of practice. Being empathetic and understanding the feelings of caregivers and clients was highlighted mainly by the employer’s group. For example, one of the participants described that not being able to understand the client’s feelings negatively affects how fresh graduates deal with their patients: “Recent graduates often neglect the client’s emotions and focus solely on performing assessments and therapy without addressing the client’s feelings and expectations. It’s important to consider this before starting the assessment.” (Employer group-1). Furthermore, patience was a trait that was highlighted by the three groups, as it is required to understand the situation and achieve results from therapy. “Being patient is key, and it’s important to remember that if you’re unsure about something, it’s best to give yourself time to gain perspective. It’s easy to jump to conclusions when you only have a limited view of the situation, so take a step back and consider the bigger picture before making any decisions.” (Alumni group-1)

**The second subtheme was adopting a flexible working approach;** participants highlighted the importance of being flexible and adaptable in adjusting or changing decisions in work: “Flexibility is crucial, particularly when working with various age groups and their caregivers. This flexibility includes adjusting the therapy approach when it doesn’t suit the patient.” (Faculty group-1).

**The third subtheme was commitment to self-development;** participants emphasised that clinicians should prioritise ongoing self-development by continuously updating and enhancing their knowledge. This includes being aware of their current skills and learning needs, taking initiative, and seeking appropriate learning opportunities. Participants from the faculty group stressed the importance of taking initiative and exploring. “Students should have a strong desire to learn, understand, explore, and take initiative in their education” (Faculty group −1). Participants from the employer group also emphasised the importance of self-motivation for development and learning. “Self-motivation is an important aspect that clinicians consider when dealing with students. Even if a student lacks prior knowledge or is apprehensive about the experience, displaying self-motivation can significantly impact how they are perceived and treated.” (Employer group – 1)

### Theme 2: Exhibiting independence in managing work.

Exhibiting independence in managing work refers to the capability of SLP graduates to manage their work responsibilities without immediate supervision.

**Recent graduates’ decision-making ability was the first subtheme** that arose under independence. Participants from the alumni group stated, “One of the most challenging skills to acquire, particularly as a recent graduate, is decision-making. It can be daunting to make decisions without supervision and support. Despite having the necessary information and skills, putting them into practice to make a sound decision is a complex process. I believe that mastering this skill is essential for everyone.” (Alumni group −1).

**The second subtheme was demonstrating analytical skills for SLPs**. Participants from the alumni group expressed this as analytical skills are essential, especially for differential diagnosis. “The ability to analyse is crucial in certain complex cases. For instance, when a patient comes in with a stroke and is confused during the evaluation session, it is important to determine whether they have aphasia, apraxia, or both. In such cases, the ability to analyse and study the case is essential and a skill that can be learned.” (Alumni group −2)

**The third subtheme involved exhibiting a strong sense of responsibility.** This was another crucial aspect of independence that all the participants expressed; for example, participants from the faculty group stated “To be a specialist who is truly dedicated means being committed to consistency, showing up for clinic sessions, prioritising the needs of patients, and continuously improving oneself.” (Faculty group −1)

Some acknowledged that though independence is important for SLP graduates, there are risks to being too independent, such as making decisions outside their expertise. The participants emphasised the need to strike a balance and receive ongoing mentorship and support from experienced professionals.

### Theme 3: Academic Knowledge for SLPs.

Participants discussed the theoretical knowledge that should be included in the SLP program to prepare students for their careers. They recognised the importance of various topics and courses already covered in the current curriculum and noted specific gaps in knowledge, discussing topics needing to be adequately covered in the academic program. Three subthemes relating to academic knowledge for SLPs were identified.

**The first subtheme was foundation knowledge essential in SLP curriculum**. Participants agreed that foundation courses in the current curriculum are fundamental to prepare students for specialised courses and clinical practice. These include courses such as anatomy, linguistics, typical development in general and speech and language development in particular. “General anatomy and physiology are some of the most important courses, whether related to speech-language pathology or not.… These subjects may seem like general knowledge initially, but as a clinician, you will rely heavily on this information.” (Alumni group −1). With regards to linguistics, a need to ensure that the content of the course applies to the Arabic language was raised by faculty members. In addition, participants indicated that learning about basic audiological assessment is essential for graduates to understand their clients better, help them make more informed decisions and communicate better with other professionals. “We all agree that learning the basics about hearing tests is essential, such as reading an audiogram, identifying the degree and causes of hearing loss.” (Faculty group −1). An additional topic that participants viewed as necessary for SLP practice is learning about cognitive functions and assessment. Concerns were raised about cognition not being given enough attention in the current program by some faculty members and employers, and discussions highlighted how general knowledge about cognition is necessary for dealing with paediatric and adult cases. Also, some participants viewed the basic principles of behavioural modification as important. Alumni and employers’ focus groups stated that many clients with communication difficulties have behavioural problems that require specific strategies for the clinician to handle the session and address their communication goals. “.. If a simple guide or program teaches us how to deal with children with these issues -referring to behavioural issues- it would be very helpful. Such a guide could include information about the types of behavioural problems that children may suffer from and how to interact with them. Often, we do not know how to handle these situations until we have undergone training courses and workshops.”(Alumni group −1)

**The second subtheme was specialised knowledge for SLP curricula**. Participants acknowledged that SLPs are expected to manage all types of communication disorders. Some participants highlighted disorders and areas that are not adequately covered in the current programme, either at a theoretical or practical level, such as swallowing disorders, voice disorders, literacy and pediatric aural rehabilitation. “I feel that paediatric and adult swallowing and voice disorders are two subjects that require more attention and are not given enough consideration.” (Employer group −2). “I learned about aural rehabilitation for adults during my studies and about paediatric aural rehabilitation during my internship, but I think the study plan should place more emphasis on paediatric.” (Alumni group −1).

**The third subtheme is Topics on current issues in SLP curricula**. Participants suggested that topics such as innovation, which reflect the evolving knowledge required in SLP, should be integrated into the curriculum. “We all understand that innovation is very important in alignment with the 2030 vision, so we need to consider, for instance, how artificial intelligence can contribute to our assessment and treatment.” (Employer group −1)

### Theme 4: General Clinical Skills for SLPs.

Participants discussed the importance of equipping recent graduates with clinical skills to prepare them for the work environment. These skills need to be developed throughout their university education until graduation. Clinical skills were grouped into two subthemes.

**The first subtheme, core skills to deliver services for clients**, refers to occupational competency within the field of SLP. This includes planning and implementing assessment and therapy, oral and written communication skills, clinical management rationale, use of evidence-based practice, and counselling.

Participants from all focus groups agreed on the importance of linking the theoretical and clinical aspects. Participants from the alumni group suggested that students should start observing clinical sessions from the 1^st^ level in the program. They talked about the difficulties they faced in the early stages of their clinical practice in integrating what they learnt and applying it to different cases with various levels of complexity. They also stated that as undergraduates, more focus was given to teaching them assessment methods than therapy techniques. “I have noticed that during lectures, …. the introduction covers the basic ideas, starting with theories, then the assessment test and its results. However, when we reach the part about therapy and therapy plans to address the disorder, there isn’t much detail provided on how to treat the disorder.” (Alumni group −1). They also suggested that teaching about disorders should consider taking a case approach so that they can learn to integrate information. “Clinical training would allow us to see disorders as whole cases and help us to focus on all aspects. For example, a post-stroke client might have swallowing, language, and speech disorders” (Alumni Focus group −1). Participants from the employers’ and faculty groups shared similar views on the importance of increasing clinical training. Interestingly, participants from the faculty group talked about the challenges they faced in covering the clinical part of the course and collaborating with hospitals and the possible alternatives, such as using simulated case discussions.

Another skill that received significant attention from all groups was communication skills in both Arabic and English languages. This includes oral and written language and non-verbal means of communication. Participants emphasised that graduates need to be competent in reporting and discussing case findings and treatment plans with team members, caregivers, and clients. “Our field depends a lot on oral and written communication, and it is very important that you can convey your message”. (Alumni focus group-1) They also discussed how graduates need to be aware of how to communicate differently when using different platforms, such as social media, for awareness campaigns, as this is becoming a part of our modern society. In addition, participants discussed the importance of counselling in SLP practice: “As a practitioner, I devote a considerable amount of time to counselling during my sessions, and sometimes the sessions are entirely devoted to counselling.” (Alumni group -1). Among the skills necessary to deliver counselling effectively is listening, as described by one of the participants. “The art of effective listening is crucial, especially when communicating with parents. It is important to refrain from interrupting and to address their inquiries attentively.” (Alumni group-1).

**The second subtheme, general work competencies for clinicians,** included skills such as teamwork, knowledge of using required software (for example, office productivity software, assessment equipment, telepractice software), and knowledge about providing services in different settings. SLPs provide services within a multidisciplinary framework, and they are expected to be able to discuss and plan the therapeutic goals of their clients with other professionals. Although being a part of a team is something that SLP students are familiar with, many of the projects were group assignments, as one of the participants in the alumni group explained. They also need to develop the skills of working with other professionals. “When working with other professionals, such as nurses or physicians, misunderstandings can sometimes arise. We need to understand our specific role and how it differs from other professions. The students must know our role and the distinctions between our profession and others.” (Alumni group −2). A suggestion that one of the faculty members gave to further the understanding of the roles and responsibilities of other professionals and how to be a member of an interdisciplinary team was to have students work on projects with students from other programs like occupational therapy or physical therapy. Another skill needed is the use of technology, which is becoming increasingly part of the required work competencies. Participants from the alumni group stressed the need to have the skill to deliver sessions through telepractice, “I think it’s essential for SLPs to know how to deliver sessions through telepractice because it’s so prevalent now. You need to understand the amount of information to share, how the session is conducted, whether it’s video or audio-only, and how to manage different age groups.” (Alumni group -1). Finally, all groups emphasised knowledge about working in different settings. “It is crucial to understand how to adjust the therapeutic goal based on the setting. For example, goal setting and documentation in long-term facilities differ from acute settings.” (Alumni group −1)

## Discussion

This study aimed to identify the skills and knowledge required of recent SLP graduates in Saudi Arabia, based on the perceptions of employers, faculty, and recent alumni. Four themes emerged from focus group discussions: Essential Individual Characteristics, Exhibiting Independence in managing work, Academic Knowledge for SLPs, and General Clinical Skills for SLPs. These themes capture generic attributes and professional knowledge and skills that underlie competency in speech-language pathology practice in Saudi Arabia. They align with the skills necessary for SLP practice and other health professions, as reported in several previous studies [7,24].

The first two themes emphasized the importance of general professional attributes, including professionalism, patience, empathy, flexibility, self-development, analytical skills, decision-making, and responsibility. These findings align with prior studies that identify such attributes as essential for a successful transition into professional practice [7]. Rather than reflecting gaps in curriculum content alone, the findings suggest that work readiness depends on the integration of personal and professional competencies developed over time. Most of these skills are fostered through both curricular and extracurricular activities, with the internship further refining these abilities, as also highlighted by Quigley et al. [25]. Nevertheless, the findings indicate a need for more structured and explicit approaches to embedding these competencies within the curriculum. Given that these qualities develop gradually, greater emphasis should be placed on targeting and systematically assessing them from the early years of the program. In line with a competency-based approach to education, it is essential to communicate clear expectations to learners [26]. With clear end goals, students can identify their strengths and areas for improvement and further develop these skills through systematic assessment strategies such as portfolios and reflective reports [27].

The third theme emphasised the essential knowledge to be delivered to SLP students. Consensus was reached on the importance of courses covering various communication disorders and foundational subjects such as anatomy, human growth and development, and linguistics. This aligns with prior research indicating that these courses effectively prepare students for working with diverse clients [28]. However, participants also identified gaps in certain content areas within communication disorders courses such as literacy, swallowing disorder, and cognition. These concerns mirror international evidence highlighting limitations in SLP graduates’ competence and confidence in specific clinical domains [8–11].The consistent gaps identified across both the current study and previous literature point to a disconnect between university training and the evolving demands of clinical practice, underscoring the need for ongoing review and adaptation of curriculum design to ensure it remains responsive to changing clinical contexts and adequately prepares graduates through sufficient foundational coverage across all communication and swallowing disorders.

A particularly important gap was identified in understanding typical Arabic language development. This limitation has direct implications for clinical competence, as the ability to distinguish between typical and disordered language development is fundamental to accurate assessment and diagnosis. This concern is supported by existing evidence indicating that only a limited number of studies, often based on small sample sizes, have examined Arabic language development, alongside a scarcity of standardized assessment tools in Arabic [29]. Consequently, the lack of robust normative data and validated tools may hinder graduates’ confidence and effectiveness in clinical decision-making. This finding extends existing literature by highlighting how linguistic and cultural factors influence work readiness, underscoring the need for contextually relevant knowledge in SLP education [30,31].

Addressing these gaps requires refining both the theoretical framework and clinical training within communication disorders courses, as aligning clinical training with theoretical knowledge is critical for enhancing training effectiveness. It also highlights the importance of fostering collaboration with clinical settings, such as hospitals, to provide targeted training opportunities.

It is important, however, to interpret these gaps within the appropriate scope of bachelor’s-level education. At this level which serves as the entry point for clinical practice in Saudi Arabia graduates are expected to develop essential foundational knowledge and skills across a broad range of communication disorders. Subspecialized clinical competency is not an expectation at graduation; rather, it is developed through clinical experience and continuing professional development. Although some study participants suggested that graduates should demonstrate competence in areas such as pediatric aural rehabilitation, these represent subspecialized competencies that go beyond the scope of entry-level training. Gaps in such areas therefore reflect the expected boundary between foundational and advanced clinical practice, rather than a shortcoming of the undergraduate program. This position is consistent with international expectations. For example, research from the United States highlights a continued need for additional training in specialized areas such as autism spectrum disorder (ASD), even following increased curricula coverage [32]. These findings reinforce the understanding that specialization is not expected at graduation but is achieved through structured post-qualification professional development. In line with this, there is a growing international emphasis on specialized training programs, and similar initiatives are emerging in Saudi Arabia including areas such as voice disorders, ASD, and swallowing disorders reflecting efforts to formalize advanced competencies through certification pathways and to support clinicians in developing expertise beyond entry-level practice. Participants also stressed the need for programs to keep up with societal advancements, highlighting the importance of incorporating contemporary topics such as innovation into SLP education. This aligns with broader calls for curricula that are adaptable and forward-looking [5]. In the Saudi context, this is particularly relevant given the rapid expansion of healthcare services and increasing demand for speech-language pathology [33].

Finally, the fourth theme encompassed general clinical skills for SLPs. Graduates raised a concern that more focus was on assessment than therapy. Similar findings were reported in the New Zealand study [7]. This may reflect the challenges faced in clinical education worldwide, as training in therapy requires access to patients and following them over a period of time. Securing this access to large numbers of students takes time and effort. Ideally, university clinics must be fully equipped and run by clinical educators with clients with various communication disorders in their caseload. Other suggestions for improvement, which can be implemented at the level of course instructors, were also provided. These included employing more case discussion-based learning and simulated-based learning. The benefits of simulated-based learning have been documented in research and shown to have a positive impact on students’ preparedness for clinical work at different levels, such as reducing anxiety, improving communication skills and increasing confidence [34–36]. Adopting these improvements and incorporating these practicum teaching approaches will help prepare graduates for the internship and work. It has to be emphasised that our graduates are expected to continue their clinical learning during the internship period, during which they will practice under the supervision of qualified clinicians. However, the practicum experience during the undergraduate years helps bridge the gap between theory and practice and prepares them gradually for independent practice.

Communication skills were identified as a core clinical competency, reflecting their central role in all aspects of SLP practice. While some studies categorize communication as a general professional skill [7], participants in this study emphasized its clinical significance, particularly in client interaction and interdisciplinary collaboration. This perspective aligns with international professional standards, such as those set by the American Speech-Language-Hearing Association, which recognize communication as a key component of clinical competence [37]. In response, some university programs have explicitly targeted communication skills within capstone courses to better prepare SLP graduates for clinical practice and client-centered care [27]. In the Saudi context, bilingual proficiency in Arabic and English further adds to the complexity of this skill, as English is widely used as the working language in healthcare settings for documentation, interdisciplinary communication, and access to clinical resources. This underscores the need for training that reflects local practice realities. Teamwork and interprofessional collaboration were identified as essential competencies, reflecting the need for SLPs to communicate effectively within multidisciplinary teams and understand the roles and boundaries of collaborating professionals. The findings highlight the importance of interprofessional collaboration as a core competency for work readiness. In response, academic programs could integrate interprofessional education (IPE) more systematically by embedding structured experiences across the curriculum, including joint case-based sessions, simulation activities, collaborative projects, and shared clinical practica with disciplines such as neurology, physical therapy, occupational therapy, and psychology. Such approaches have been shown to strengthen students’ understanding of other health professionals’ roles and provide authentic opportunities to apply teamwork and communication skills, ultimately enhancing graduates’ readiness for collaborative healthcare practice [38]. Moreover, participants discuss the necessity of exposing SLP graduates to various work settings. Currently, the SLP program in Saudi Arabia primarily focuses on preparing graduates to work in hospital settings, as the internship or clinical placement is predominantly carried out in hospitals. However, it is essential also to equip graduates to work in settings such as long-term facilities or schools to provide them with a broader range of career opportunities. Challenges related to school-based SLPs primarily stem from limited job prospects and a need for an understanding of the role of SLPs within the system. Fortunately, speech-language services in Saudi Arabia are expanding, underscoring the need to modify the curriculum to encompass training for multiple settings, not just hospitals.

### Recommendations and Implications

Overall, the findings of the present study highlight several practical areas for further enhancement. From an educational perspective, the findings emphasize the importance of ongoing curriculum review and adaptation to ensure sufficient foundational coverage across communication and swallowing disorders, particularly in areas identified as requiring further emphasis, such as literacy, swallowing disorder, and cognition, while also ensuring that programs remain responsive to societal and professional changes. The findings also highlight the importance of further strengthening the alignment between theoretical knowledge and clinical practice through the incorporation of more simulation-based and case-based learning approaches, as well as increasing students’ exposure to diverse clinical settings. Greater emphasis should also be placed on the systematic development of personal and professional competencies from the early years of training through explicit learning expectations and structured assessment approaches, such as reflective reports and portfolios. In clinical practice, the findings suggest the importance of continuing to build graduates’ confidence not only in assessment, but also in delivering intervention. Strengthening communication skills and interprofessional collaboration is essential. This may be supported by embedding structured interprofessional learning experiences, such as joint case discussions and shared clinical practica with other healthcare disciplines, to further develop teamwork and communication skills required in multidisciplinary practice. From a research perspective, the findings underscore the need to expand contextually relevant Arabic resources, including normative data on typical Arabic language development and standardized assessment tools. Strengthening collaboration between linguists and SLPs may further support the development of culturally and linguistically appropriate clinical resources and enhance graduates’ preparedness for professional practice. In addition, future research should explore the impact of curriculum and training changes on workforce readiness and graduate preparedness over time.

### Study limitations

Several limitations should be acknowledged to guide future research in gaining a more comprehensive understanding of the knowledge, clinical skills, and attitudes required to develop proficient SLPs working with individuals with communication disorders in Saudi Arabia. First, our sample primarily consisted of female SLPs and students, which may reflect the predominance of females in the field [39]. However, future research would benefit from including male SLPs and students to allow for a comparative analysis of perspectives. Second, all student and academic focus group participants were recruited from a single university, which may limit the generalizability of our findings. Expanding the study to multiple institutions would provide a broader perspective. Finally, since the interviewers were faculty members in the Speech and Hearing program, there is a possibility of social desirability bias, where participants may have felt hesitant to share negative experiences or critical viewpoints. While we emphasised that the study aimed to enhance educational outcomes and assured participants that their responses would have no negative consequences, we cannot entirely rule out this potential influence. However, efforts were made to encourage open and honest discussions, allowing participants to express essential perspectives. Despite these limitations, the study provides valuable insights into the current educational landscape for SLPs in Saudi Arabia, laying the foundation for future research and program development.

## Conclusion

In conclusion, this study identified the essential skills and knowledge required for recent SLP graduates in Saudi Arabia based on the perspectives of employers, faculty members, and recent graduates. Four key themes emerged essential individual characteristics, exhibiting independence in managing work, academic knowledge for SLPs, and general clinical skills for SLPs which align with international expectations for SLP and allied health practice. The findings highlight the importance of developing personal and professional attributes alongside foundational knowledge and clinical competencies. They also emphasize the need to strengthen preparation in selected clinical areas, expand contextually relevant Arabic resources, and continue adapting SLP curricula to evolving professional and societal demands. Overall, as speech-language pathology services continue to expand in Saudi Arabia, educational programs must continue to evolve to ensure graduates are prepared for increasingly diverse clinical roles and practice settings.

## Supporting information

S1 Appendix The interview guide. (DOCX)

S2 Appendix The codebook. (DOCX)
