# Supplementary material for: Qualitative investigation of skills and knowledge required for job readiness in newly graduated speech-language pathologists
Source: PLoS One. 2026 Jul 21;21(7):e0354088. doi: 10.1371/journal.pone.0354088 (PMC13387538; doi:10.1371/journal.pone.0354088)
Supplement: S1 Appendix — (DOCX) [file pone.0354088.s001.docx]

**Appendix (1)**

**The Interview Guide**

**Faculty Group**

1. What are the most critical personal skills a Speech-Language Therapy (SLT) graduate should possess? (Examples of abilities: teamwork)
   - Based on your experience in delivering this program, what personal skills still need to be addressed by the program but are required by graduates in SLT?
2. What are the most critical sciences, information, and knowledge that an SLT graduate should have? (Examples of knowledge include anatomical knowledge, physiological knowledge, linguistic knowledge, and normal and abnormal processes in communication)
   - Based on your experience in delivering this program, what information and knowledge are not provided by the program that is important to offer/deliver?
3. What are the most critical areas an SLT graduate should be familiar with regarding the workplace (Examples: school/rehabilitation centre) and the types of disorders they may encounter (Examples: autism)?
   - Based on your experience in delivering this programme, what areas does the programme need to cover for graduates in SLT?
4. What is the current demand in the job market for SLT graduates?
   - How do you believe the current demand in the job market is determined?
5. What are the most critical requirements and developments in the modern job market in Saudi Arabia concerning SLT specialities that should be considered in undergraduate programmes for programme graduates to compete with those from developed countries?
6. How do you see the evolution of the SLT profession in the next ten years?
   - How do you think the expected developments will affect the skills needed by future graduates?
7. What teaching or training methods do you suggest should be used to help graduates develop their personal skills?
8. What teaching or training methods should be used to help graduates develop their cognitive and clinical skills?
9. Do you suggest adding a new course to the programme that is important to offer during the study phase of the SLT?
10. What qualities does the job market seek when hiring graduates in speech-language pathology/audiology?
11. What areas or tasks should graduates expect to be familiar with upon graduation?
12. What do you think is the most suitable title for the SLT programme? (Options: communication and swallowing disorders therapy, speech and language and swallowing disorders, communication disorders).

**Employee/ Clinician Group**

1. What essential personal skills should a Speech-Language Therapy (SLT) graduate possess? (Examples of abilities: teamwork)
   - Based on your experience working with graduates from this program, what personal skills still need to be addressed by the program but are required by graduates in SLT?
2. What are the most critical sciences, information, and knowledge that an SLT graduate should have? (Examples of knowledge include anatomical knowledge, physiological knowledge, linguistic knowledge, and normal and abnormal processes in communication)
   - Based on your experience working with graduates from this program, what information and knowledge are not provided by the program that is important to offer/deliver?
3. What are the most critical areas an SLT graduate should be familiar with regarding the workplace (Examples: school/rehabilitation centre) and the types of disorders they may encounter (Examples: autism)?
   - Based on your experience working with graduates from this program, what areas does the programme need to cover for graduates in SLT?
4. What is the current demand in the job market for SLT graduates?
   - How do you believe the current demand in the job market is determined?
5. What are the most critical requirements and developments in the modern job market in Saudi Arabia concerning SLT specialities that should be considered in undergraduate programmes for programme graduates to compete with those from developed countries?
6. How do you see the evolution of the SLT profession in the next ten years?
   - How do you think the expected developments will affect the skills needed by future graduates?
7. What teaching or training methods do you suggest should be used to help graduates develop their personal skills?
8. What teaching or training methods should be used to help graduates develop their cognitive and clinical skills?
9. Do you suggest adding a new course to the programme that is important to offer during the study phase of the SLT?
10. What qualities does the job market seek when hiring graduates in speech-language pathology/audiology?
11. What areas or tasks should graduates expect to be familiar with upon graduation?
12. What do you think is the most suitable title for the SLT programme? (Options: communication and swallowing disorders therapy, speech and language and swallowing disorders, communication disorders).

**Alumni Group**

1. What are the most essential personal skills a Speech-Language Therapy (SLT) graduate should possess? (Examples of abilities: teamwork)

- Based on your experience, what personal skills are not targeted by the programme but are needed by a graduate in SLT?

1. What are the most critical sciences, information, and knowledge that an SLT graduate should have? (Examples of knowledge include anatomical knowledge, physiological knowledge, linguistic knowledge, and normal and abnormal processes in communication)
   - Based on your experience, what information and knowledge are not provided by the program that is important to offer/deliver?
2. What are the most critical areas an SLT graduate should be familiar with regarding the workplace (Examples: school/rehabilitation centre) and the types of disorders they may encounter (Examples: autism)?
   - Based on your experience, what areas does the programme need to cover for graduates in SLT?
3. What is the current demand in the job market for SLT graduates?
   - How do you believe the current demand in the job market is determined?
4. What are the most critical requirements and developments in the modern job market in Saudi Arabia concerning SLT specialities that should be considered in undergraduate programmes for programme graduates to compete with those from developed countries?
5. How do you see the evolution of the SLT profession in the next ten years?
   - How do you think the expected developments will affect the skills needed by future graduates?
6. What teaching or training methods do you suggest should be used to help graduates develop their personal skills?
7. What teaching or training methods should be used to help graduates develop their cognitive and clinical skills?
8. Do you suggest adding a new course to the programme that is important to offer during the study phase of the SLT?
9. What qualities does the job market seek when hiring graduates in speech-language pathology/audiology?
10. What are the job market expectations for entry-level roles during the training phase?
    - Do you believe the job market demands knowledge in areas where graduates may lack preparedness?
11. Based on your experience, what is the most crucial lesson you learned in university?
    - Were there specific courses that had a significant impact on your career? Or were there particular skills that you honed during your time in university?
12. What do you think is the most suitable title for the SLT programme? (Options: communication and swallowing disorders therapy, speech and language and swallowing disorders, communication disorders).
