# Supplementary material for: Qualitative investigation of skills and knowledge required for job readiness in newly graduated speech-language pathologists
Source: PLoS One. 2026 Jul 21;21(7):e0354088. doi: 10.1371/journal.pone.0354088 (PMC13387538; doi:10.1371/journal.pone.0354088)
Supplement: S2 Appendix — (DOCX) [file pone.0354088.s002.docx]

**Appendix (2): The Codebook**

| Code | Meaning | Example |
| --- | --- | --- |
| Multitasking | Learn how to focus on multiple tasks during a single session. | “Specialists working with paediatrics should learn to multitask - listen, observe, record and interact simultaneously.” (alumni focus group 1) |
| Typical communication development | The communication milestones of children in acquiring language and speech. | “Parents often have concerns about their child's language and speech development milestones, so it's important to know what's typical for the pediatric population.” (alumni focus group 1) |
| Patience | Having patience during rehabilitation is crucial as therapy takes time to show benefits and requires consistent practice. | "As clinicians, it's important for them to practice patience, as the outcome may take time to be evident." (employer focus group 2) |
| Teamwork | learning how to collaborate with professionals from various fields, such as occupational therapy, nursing, and medicine. | Interdisciplinary practice is important but not commonly taught in university. While some students attempt to collaborate, there aren't enough true interdisciplinary learning opportunities. For example, a course could focus on common stroke patient issues, where students from different disciplines work together towards shared goals.  (Faculty focus group) |
| Cognition | Understanding the cognitive ability | “When we were studying, we did not know the role of Speech and Language Pathologists in cognitive intervention, especially in severe cases for both paediatric and adult cognitive ability.” (Employer focus group 1)  Or “Cognitive abilities form the basis for most language skills, so it is imperative that we introduce cognition as a core course for undergraduate students.” (Faculty focus group) |
| Oral/written communication | Discussing the importance of effective communication involves active listening and proficiency in both oral and written forms, including email and report writing. | “Clinician/student should have the ability introducing oneself to the family, explaining one's role clearly, and using simple language to provide education.” (Faculty focus group). |
| Empathy | Reflecting the importance to understands caregiver or client feelings | “Sometimes, caregivers come to the session in denial that their loved one has a communication disorder. Therefore, it's important to understand their feelings to know how to respond.” (Employer focus group 1) |
| Different setting | Discus the different work setting (e.g. Hospital/school) and different service delivery method (in person or remotely) | “It is important to understand that each field of work has its own nature and expectations. For instance, the educational field differs from the medical field and private clinics. Therefore, it is essential to know the specifics of each field, including the kinds of cases you may encounter and the necessary knowledge you need to possess. I have noticed that the educational field requires a distinct set of skills and knowledge.” (Alumni focus group 2) |
